# Supplementary material for: IL-18 favors Th2 responses in sporotrichosis caused by Sporothrix globosa, prolonging the course of the disease
Source: PLoS Negl Trop Dis. 2025 Jun 9;19(6):e0013170. doi: 10.1371/journal.pntd.0013170 (PMC12173405; doi:10.1371/journal.pntd.0013170)
Supplement: S1 Appendix — (DOCX) [file pntd.0013170.s001.docx]

**Supporting materials and methods**

**Quantitative real-time PCR**

Quantitative real-time PCR (qPCR) was employed to characterize the mRNA expression of different genes. First, total RNA was isolated using M5 Universal RNA Mini Kit (Mei5bio, Beijing, China) and was normalized to 1 μg. Second, cDNA was synthesized with the All-in-One 5X RT MasterMix (abm, Canada) according to the manufacturer’s instructions. Furthermore, the detection of all target genes and the control, GAPDH, was performed using BlasTaq 2X qPCR MasterMix (abm, Canada), and all data were analyzed using the 2 ^−ΔΔt^ method.

**Immunohistochemistry**

Fresh skin specimens, including sporotrichosis skin tissue and normal skin tissue, as well as normal lymph nodes, were prepared as formaldehyde-fixed and paraffin-embedded (FFPE) sections with a thickness of 4 μm. Following deparaffinization and rehydration, the paraffin sections underwent antigen retrieval and were subsequently blocked with 3% hydrogen peroxide for 15 min. Subsequently, they were incubated with ready-to-use goat serum (AR0009, BOSTER) for 30 min at 37℃. Next, the primary antibodies (clone, manufacturer, and working concentration as listed in Table 3) were added and incubated overnight at 4℃. Appropriate negative (no primary antibody) and positive (normal lymph nodes) controls were used in parallel with each set. The slides were washed with PBST three times and then incubated with HRP-conjugated affinipure secondary antibody for 1 hour at room temperature. Following three PBST washes, the specimens were stained with DAB (AR1027, BOSTER). Subsequently, the samples underwent cleaning and were counterstained with hematoxylin. IHC images were visualized under an Olympus BX53 upright microscope, with five random fields acquired per section. To analyze the correlation between various cytokines, serial sections of the same tissue were stained and visual fields were selected at the same location.

**Multiplexed immunohistochemistry (mIHC) assay**

The mIHC assay was performed according to the manufacturer’s instructions (abs50013, absin), slides (4μm) were used for staining. In brief, the tissues were dewaxed and rehydrated before antigen retrieval. Slides were then washed with TBST and blocked with ready-to-use goat serum (AR0009, BOSTER) at 37 ℃ for 30 min. The primary antibody (clone, manufacturer and working concentration were listed in Table 3) was incubated overnight at 4 ℃ and the secondary antibody (HRP) incubation was at room temperature for 15 min. The slides were then repeated for the antigen retrieval and above antibody incubation process. The nuclei were stained with DAPI before imaging. Slides were imaged using a Nikon AXR（Ti2-E）confocal laser scanning microscope with three representative fields systematically captured per specimen.

**Flow Cytometry**

Cells were analyzed with a 13-color flow cytometer (FACSAria III, BD Biosciences, Franklin Lakes, NJ). In vitro in order to detect IFN-γ, IL-4, IL-17A and IL-2 expressions, PBMCs were first treated with Leukocyte Activation Cocktail, with BD GolgiPlug (550583, BD, San Jose, CA, USA) at 37°C, 5% CO2 for 4 h in vitro. Then, the cells were stained with LIVE/DEAD Fixable Aqua (L34966, Invitrogen) for 30 minutes at 4°C, followed by anti-human CD4-FITC (Cat. No.11-0049-42, clone RPA-T4, eBioscience), anti-human CD8-PerCP-Cy5.5 (Cat. No. 45-0088-42, clone RPA-T8, eBioscience), anti-human IL-18Rα-PE (Cat. No. 12-7183-42, clone H44, eBioscience) or anti-human CD4-PE (Cat. No. 12-0049-42, clone RPA-T4, eBioscience) for another 30 minutes at 4°C. Subsequently, the cells were fixed and permeabilized using the Cytofix/Cytoperm™Fixation/Permeabilization Kit (Cat. No. 554714, BD Pharmingen), and treated with anti-human IL-4 PerCP-Cy5.5 (Cat. No. 561234, clone 8D4-8, BD Pharmingen) or anti-human IL-4-PE (Cat. No. 12-7049-42, clone 8D4-8, eBioscience), anti-human IL-17A-APC (Cat. No. 17-7179-41, clone eBlo64DEC17, eBioscience), anti-human IFN-γ-PerCP-Cy5.5 (Cat. No. 45-7319-42, clone 4S.B3, eBioscience), as well as anti-human IL-2-PE (Cat. No. 560709, clone MQ1-17H12, BD Pharmingen) in accordance with the manufacturer's instructions.
