# Supplementary material for: IL-18 favors Th2 responses in sporotrichosis caused by Sporothrix globosa, prolonging the course of the disease
Source: PLoS Negl Trop Dis. 2025 Jun 9;19(6):e0013170. doi: 10.1371/journal.pntd.0013170 (PMC12173405; doi:10.1371/journal.pntd.0013170)
Supplement: S1 Table — (DOCX) [file pntd.0013170.s002.docx]

**S1 Table. Clinical characteristics of patients enrolled**

| **Patient NO.** | **Sex** | **Age (year)** | **Clinical Form** | **Disease duration**  **(month)** | **Location** |
| --- | --- | --- | --- | --- | --- |
| 1 | F | 52 | Fixed | 2 | hand |
| 2 | M | 51 | Fixed | 4 | hand |
| 3 | F | 52 | Lymphocutaneous | 10 | upper limb |
| 4 | F | 49 | Fixed | 3.5 | face |
| 5 | F | 55 | Lymphocutaneous | 12 | upper limb |
| 6 | F | 48 | Lymphocutaneous | 2 | forearm |
| 7 | M | 69 | Fixed | 3 | face |
| 8 | M | 67 | Lymphocutaneous | 10 | upper limb |
| 9 | F | 60 | Fixed | 5 | hand |
| 10 | F | 49 | Fixed | 12 | hand |
| 11 | F | 52 | Lymphocutaneous | 2 | face |
| 12 | F | 61 | Lymphocutaneous | 1 | upper limb |
| 13 | F | 65 | Lymphocutaneous | 3 | face |
| 14 | M | 67 | Fixed | 9.5 | neck |
| 15 | F | 62 | Lymphocutaneous | 4 | hand |
| 16 | F | 64 | Lymphocutaneous | 6 | forearm |
| 17 | F | 67 | Fixed | 9 | forearm |
| 18 | M | 56 | Lymphocutaneous | 18 | face |
| 19 | M | 67 | Fixed | 10 | face |
| 20 | M | 70 | Lymphocutaneous | 24 | hand |
| 21 | F | 48 | Fixed | 2 | hand |
| 22 | F | 65 | Fixed | 8 | forearm |
| 23 | M | 53 | Lymphocutaneous | 3.5 | upper limb |
| 24 | F | 67 | Fixed | 8 | forearm |
| 25 | F | 58 | Fixed | 2 | hand |
| 26 | M | 49 | Fixed | 12 | ear |
| 27 | F | 64 | Fixed | 7 | lower leg |
| 28 | F | 69 | Lymphocutaneous | 2 | shoulder |
| 29 | F | 58 | Lymphocutaneous | 12 | lower limb |
| 30 | F | 53 | Fixed | 4.5 | hand |
| 31 | F | 52 | Lymphocutaneous | 10 | upper limb |
| 32 | M | 43 | Fixed | 2 | face |
| 33 | F | 50 | Fixed | 3.5 | hand |
| 34 | M | 49 | Lymphocutaneous | 4 | face |
| 35 | M | 64 | Lymphocutaneous | 3 | lower limb |
| 36 | F | 64 | Lymphocutaneous | 6 | forearm |
| 37 | F | 59 | Fixed | 18 | neck |
| 38 | M | 54 | Fixed | 3 | hand |
| 39 | M | 67 | Lymphocutaneous | 6 | face |
| 40 | F | 52 | Lymphocutaneous | 1 | foot |
| 41 | F | 46 | Fixed | 1 | face |
| 42 | M | 61 | Lymphocutaneous | 5 | neck |
| 43 | F | 49 | Lymphocutaneous | 7 | upper limb |
| 44 | M | 48 | Fixed | 10 | chest |

F: female; M: male.
