# Supplementary material for: IL-18 favors Th2 responses in sporotrichosis caused by Sporothrix globosa, prolonging the course of the disease
Source: PLoS Negl Trop Dis. 2025 Jun 9;19(6):e0013170. doi: 10.1371/journal.pntd.0013170 (PMC12173405; doi:10.1371/journal.pntd.0013170)
Supplement: S2 Table — (DOCX) [file pntd.0013170.s003.docx]

**S2 Table. Antibodies and cytokines used in cell culture, IHC, mIHC, and WB.**

| **Antibody/Cytokines** | **Vendor** | **NO./Clone** | **Working concentration** |
| --- | --- | --- | --- |
| IL-18 antibody | R&D Systems | Cat# AF2548 | 1.2 µg/mL |
| IL-2 antibody | R&D Systems | Cat# AF-202-SP | 1 µg/mL |
| BAY 11-7082 | MCE | Cat# HY-13453 | 5 μM |
| Recombinant human IL-18 protein | R&D Systems | Cat# 9124-IL | 0, 50, 100 ng/mL |
| Recombinant human IL-2 protein | R&D Systems | Cat# 202-IL | 10 ng/mL |
| IL-18 Rabbit mAb | Abcam | ab243091/EPR19954-188 | IHC-P 1:500  mIHC 1:1000  WB 1:1000 |
| Caspase-1 Rabbit mAb | CST | #P29466/E4R8K | IHC-P 1:500 |
| Cleaved Caspase-1 Rabbit mAb | CST | #4199/D57A2 | WB 1:1000 |
| IL-2 Rabbit mAb | Abcam | ab52632/EP1347Y | IHC-P 1:400 |
| IL-4 Mouse mAb | Abcam | ab239508/C1 | IHC-P 1:200 |
| IL-17A Rabbit pAb | Abcam | ab79056 | IHC-P 1:400 |
| IFN-γ Mouse mAb | Abcam | ab218426/IFNG/466 | IHC-P 1:200 |
| CD11c Rabbit mAb | Abcam | ab52632/EP1347Y | IHC-P 1:500 |
| CD68 Rabbit mAb | Abcam | ab213363/EPR20545 | mIHC 1:1000 |
| Langerin Rabbit mAb | Abcam | ab192027/EPR15863 | mIHC 1:1000 |
| CD3 Rabbit mAb | Abcam | ab16669/SP7 | mIHC 1:150 |
| GAPDH Rabbit mAb | CST | # 5174/D16H11 | WB 1:1000 |
| Goat anti-rabbit pAb | Abcam | ab205718 | IHC-P 1:6000  WB 1:10000 |
| Goat anti-mouse pAb | Abcam | ab205719 | IHC-P 1:6000 |
