# Supplementary material for: IL-18 favors Th2 responses in sporotrichosis caused by Sporothrix globosa, prolonging the course of the disease
Source: PLoS Negl Trop Dis. 2025 Jun 9;19(6):e0013170. doi: 10.1371/journal.pntd.0013170 (PMC12173405; doi:10.1371/journal.pntd.0013170)
Supplement: S3 Table — (DOCX) [file pntd.0013170.s004.docx]

**S3 Table. Primers for qPCR.**

| **Gene** | **Genbank accession** | **Forward primer and Reverse primer (5′→3′)** | **Product size**  **（bp）** |
| --- | --- | --- | --- |
| IL-18 | [XM_054368670.1](https://www.ncbi.nlm.nih.gov/entrez/viewer.fcgi?db=nucleotide&id=2462524982) | F: 5’-CTGCCACCTGCTGCAGTCTA-3’  R: 5’-TCTACTGGTTCAGCAGCCATCTTTA-3’ | 143 |
| Caspase-1 | [XM_054370130.1](https://www.ncbi.nlm.nih.gov/entrez/viewer.fcgi?db=nucleotide&id=2462527974) | F: 5’-TGCTCTTCCACACCAGATAATGT-3’  R: 5’-TCCACATCACAGGAACAGGC-3’ | 110 |
| IL-18R | [XM_047446169.1](https://www.ncbi.nlm.nih.gov/entrez/viewer.fcgi?db=nucleotide&id=2217331778) | F: 5’-TCTGAAGTGGAAGGCCGATA-3’  R: 5’-CACAGCGCAATCTTTAGTCTCA-3’ | 313 |
| IL-18BP | [NM_005699.3](https://www.ncbi.nlm.nih.gov/entrez/viewer.fcgi?db=nucleotide&id=323098347) | F: 5’-TTGAGCCTGAAGTGGTCCAACT-3’  R: 5’-TCTAATCTACCCCCATGCCACC-3’ | 240 |
| IL-2 | [NM_000586.4](https://www.ncbi.nlm.nih.gov/entrez/viewer.fcgi?db=nucleotide&id=1777425429) | F: 5’-TACATGCCCAAGAAGGCCAC-3’  R: 5’-TTGCTGATTAAGTCCCTGGGT-3’ | 131 |
| GAPDH | [NM_001256799.3](https://www.ncbi.nlm.nih.gov/entrez/viewer.fcgi?db=nucleotide&id=1676318038) | F: 5’-GCACCGTCAAGGCTGAGAAC-3’  R: 5’-TGGTGAAGACGCCAGTGGA-3’ | 138 |
