# Supplementary figures and images for: IL-18 favors Th2 responses in sporotrichosis caused by Sporothrix globosa, prolonging the course of the disease
Source: PLoS Negl Trop Dis. 2025 Jun 9;19(6):e0013170. doi: 10.1371/journal.pntd.0013170 (PMC12173405; doi:10.1371/journal.pntd.0013170)

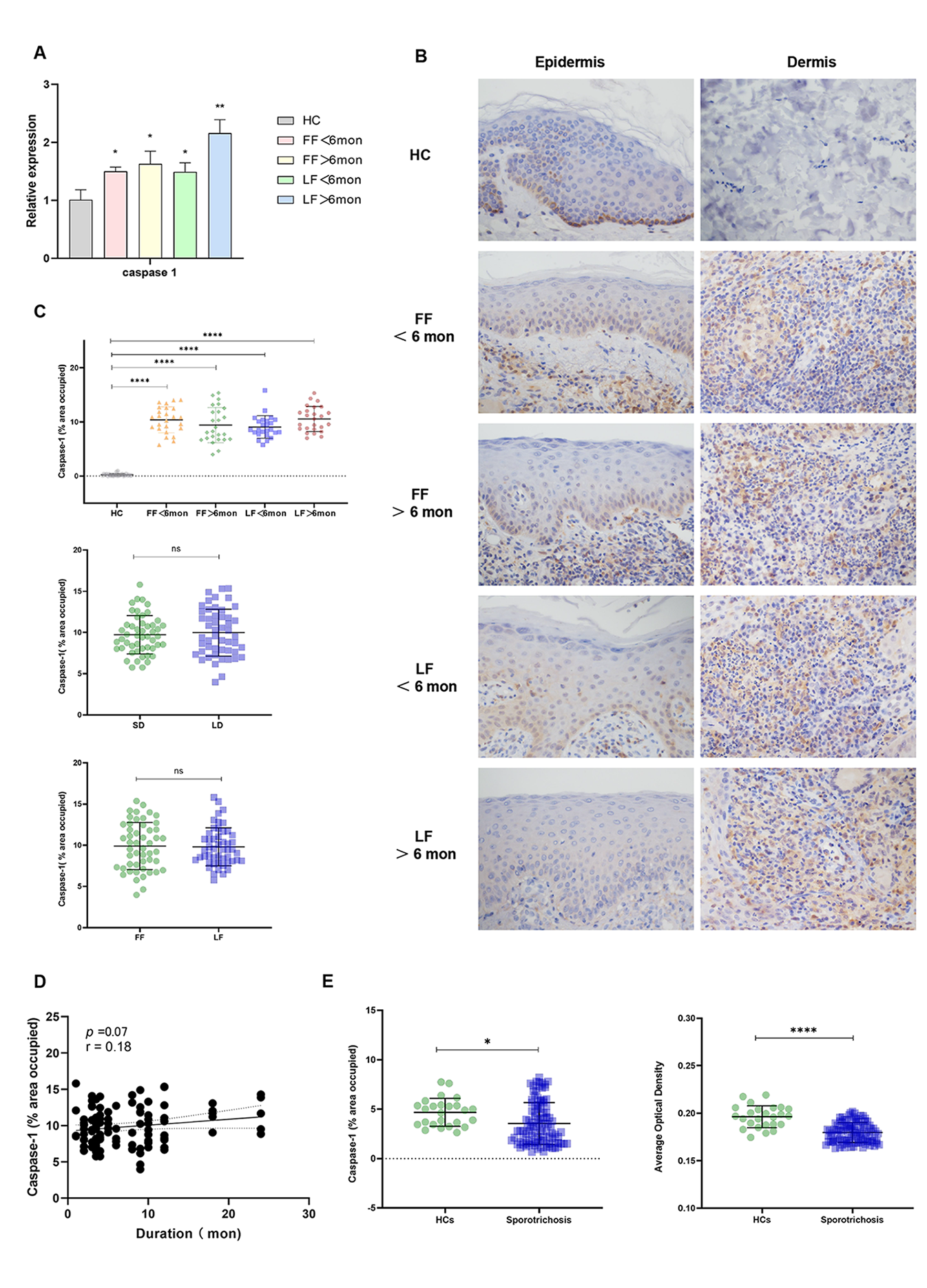

Supplement: S1 Fig — (A) qPCR analysis for mRNA expression of caspase-1 in HC skins and sporotrichosis lesional skins. (B) Representative IHC images for caspase-1 expression in each group. Original magnification ×400. (C) Statistical analysis of caspase-1 distribution area in the dermis of HC skin and sporotrichosis lesional skin (above); with comparisons between LD and SD (middle); between FF and LF (below). (D) Correlation between dermal caspase-1 expression and disease duration. (E) Statistical analysis of caspase-1 distribution area and average optical density (AOD) in the epidermis of HC skins and sporotrichosis lesional skins. Sample sizes: HC: n = 5, lesion: n = 20, n = 5 per subgroup. The data shown are expressed as mean ± SD of three independent experiments, and the measured values from individual patients were plotted by dots. The graphs showed a linear fit for patients. The data were analyzed with Pearson correlation analysis. The dotted line demonstrated a 95% confidence interval; Pearson’s correlation coefficient (r) and associated P values are shown on the graph. *P < 0.05, **P < 0.01, ***P < 0.001, ****P < 0.0001, and ns P > 0.05. (TIF) [file pntd.0013170.s006.tif]

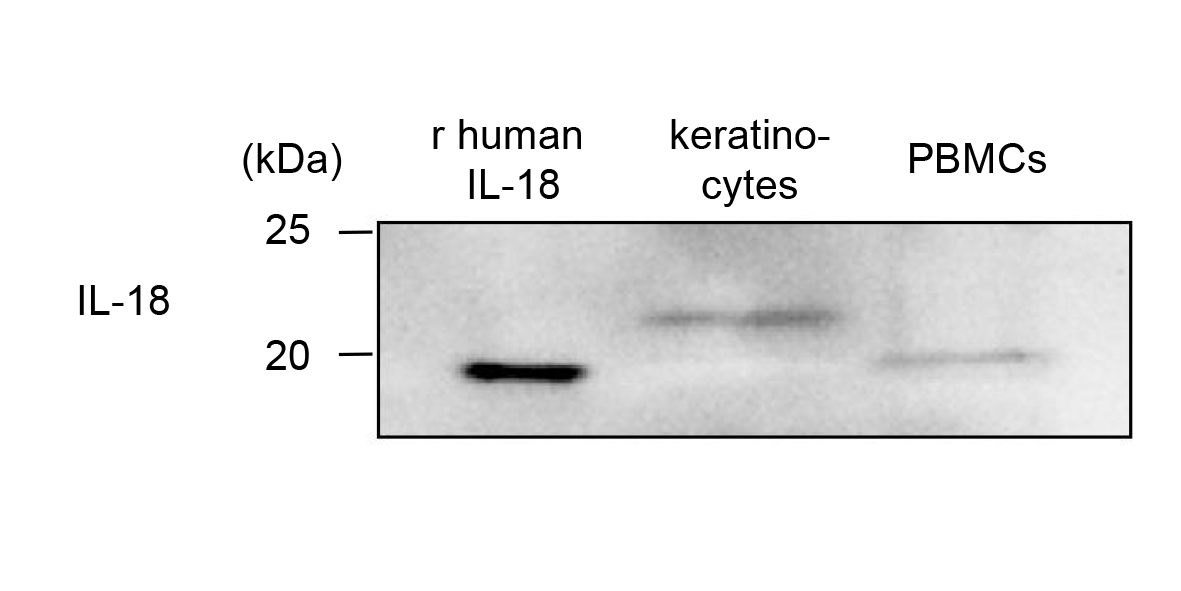

Supplement: S2 Fig — Western blot analysis of IL-18 expression in keratinocytes and PBMCs after S. globosa stimulation. Lane 1: recombinant human IL-18; lane 2: human primary keratinocytes lysates; lane 3: PBMCs lysates. The lysates were from three independent experiments. (TIF) [file pntd.0013170.s007.tif]

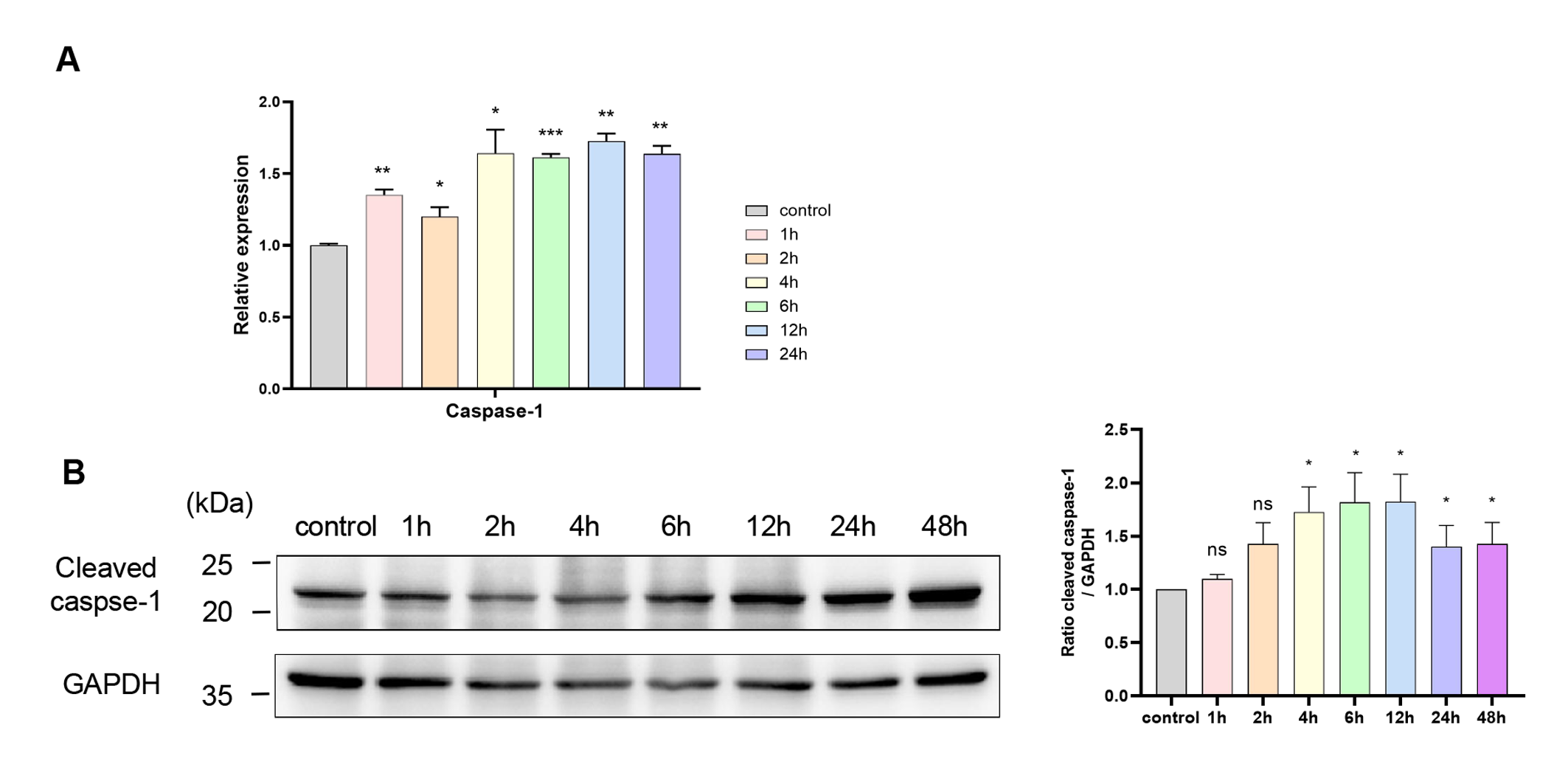

Supplement: S3 Fig — (A) The mRNA levels of caspase-1 in PBMCs after S. globosa stimulation (MOI = 5) at different time points. (B) Western blot analysis of cleaved caspase-1 expression in PBMCs after S. globosa stimulation (MOI = 5) at different time points (left). Statistical analysis of relative protein expression was quantified with ImageJ software (right). The data shown are expressed as mean ± SD of three independent experiments. *P < 0.05, **P < 0.01, ***P < 0.001, ****P < 0.0001, and ns P > 0.05. (TIF) [file pntd.0013170.s008.tif]

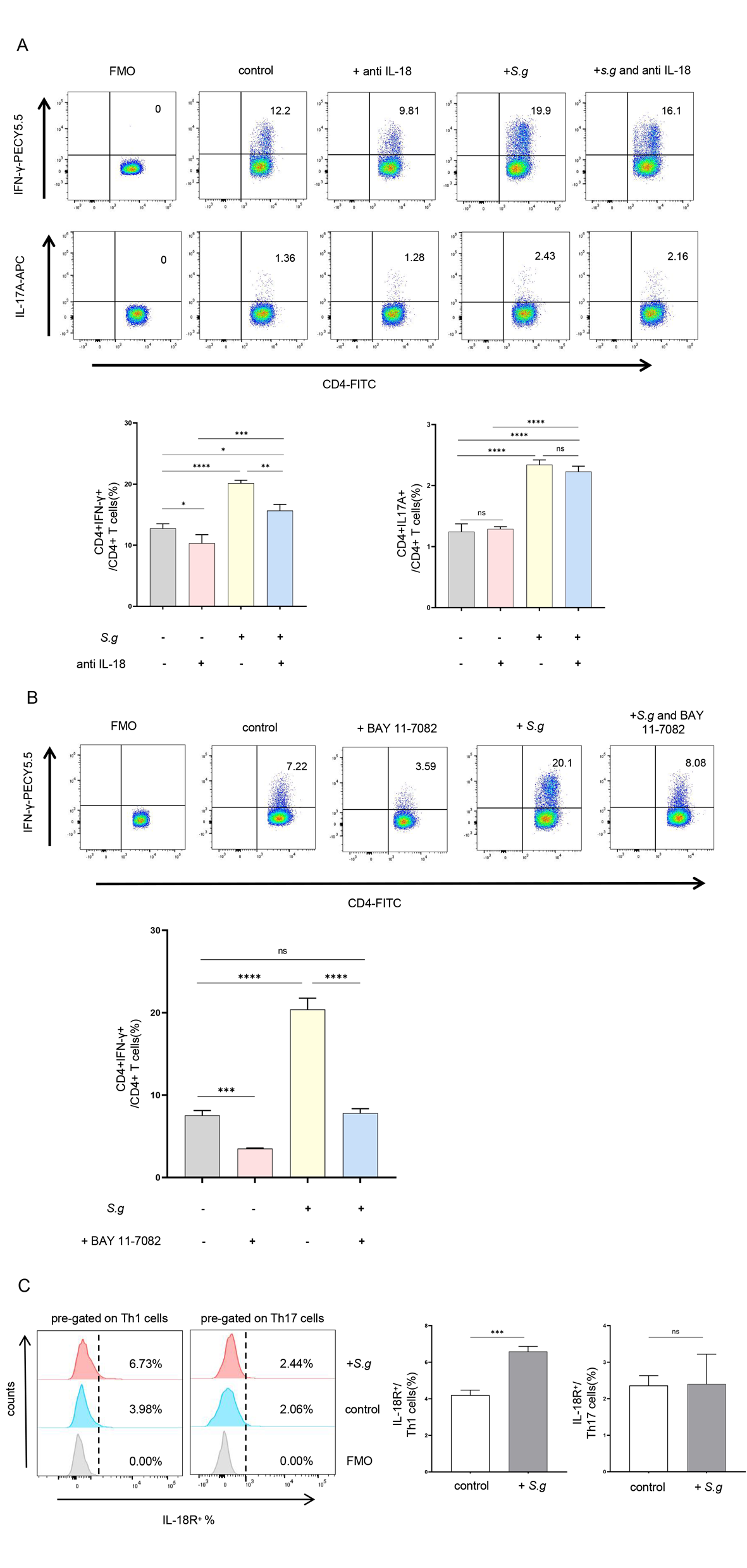

Supplement: S4 Fig — (A, B) Effect of neutralizing IL-18 (A) and blocking NF-κB (B) on CD4+IFN-γ+ Th1 and CD4+IL-17A+ Th17 immune responses during S. globosa stimulation, analyzed by flow cytometry. (C) The expression of IL-18Rα in CD4+IFN-γ+ Th1 and CD4+IL-17A+ Th17 cells. The data shown are expressed as mean ± SD of three independent experiments. *P < 0.05, **P < 0.01, ***P < 0.001, ****P < 0.0001 and ns P ≥ 0.05. (TIF) [file pntd.0013170.s009.tif]

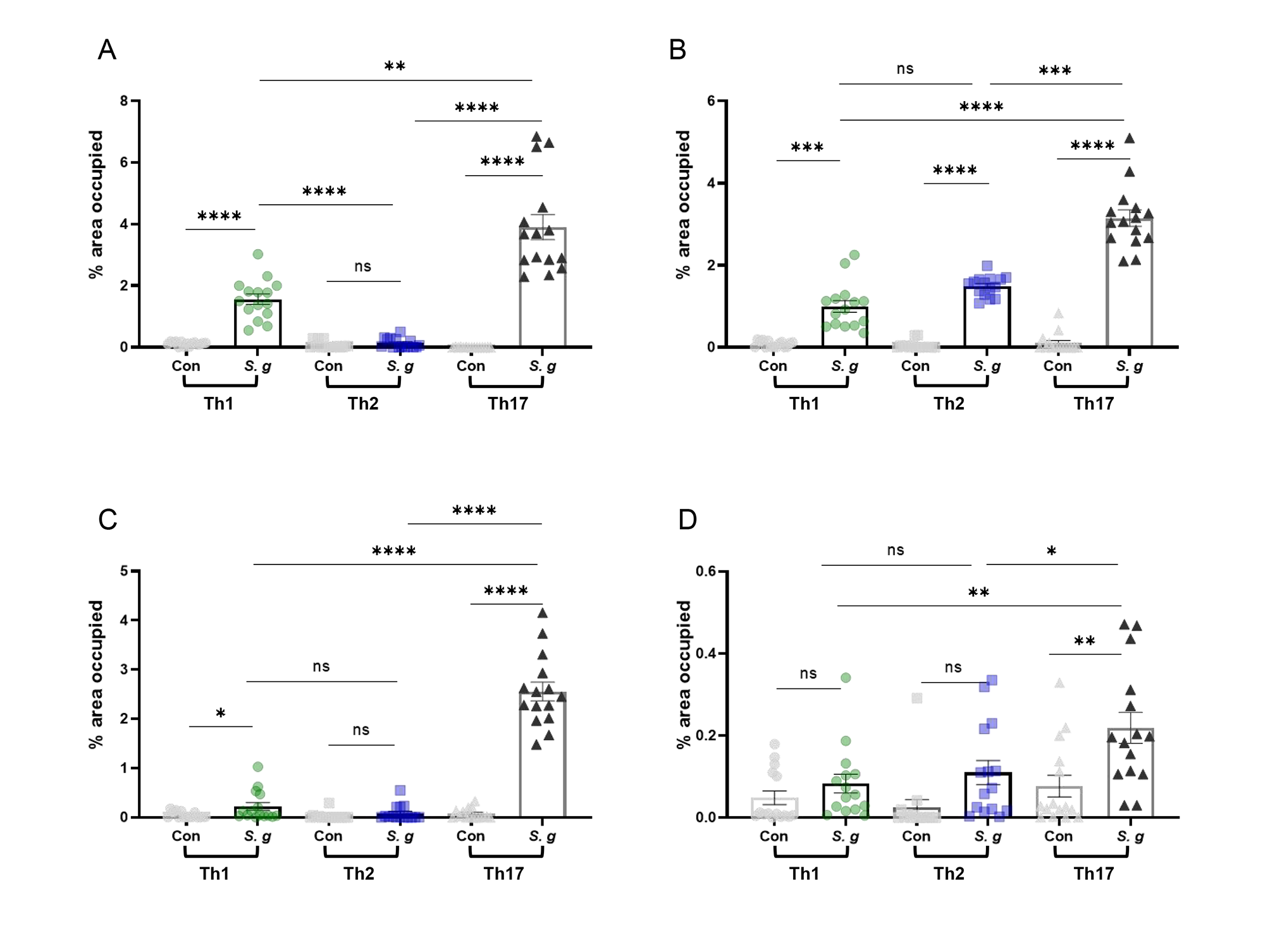

Supplement: S5 Fig — (A-D) Cytokine profiles (IFN-γ/IL-4/IL-17A) quantified by immunohistochemistry at 1, 2, 4, and 5 weeks post-injection of 1 × 10⁷ heat-killed S. globosa yeast cells (6–8-week-old BALB/c mice; PBS-injected controls). Individual data points from all mice are displayed, with results presented as mean ± SD. *P < 0.05, **P < 0.01, ***P < 0.001, ****P < 0.0001 and ns P ≥ 0.05. Con, control. (TIFF) [file pntd.0013170.s010.tiff]
