# Supplementary material for: IL-18 favors Th2 responses in sporotrichosis caused by Sporothrix globosa, prolonging the course of the disease
Source: PLoS Negl Trop Dis. 2025 Jun 9;19(6):e0013170. doi: 10.1371/journal.pntd.0013170 (PMC12173405; doi:10.1371/journal.pntd.0013170)
Supplement: Attachment — Submitted filename: Response to Reviewers.docx [file pntd.0013170.s012.docx]

**Dear Editor and Reviewers,**

Thank you for your thorough review of our manuscript "IL-18 favors Th2 responses in sporotrichosis caused by *Sporothrix globosa*, prolonging the course of the disease [PNTD-D-25-00591]" and for providing valuable feedback that has significantly improved the quality of our work. We have carefully addressed all comments raised by the reviewers and made corresponding revisions to the manuscript. Below, we provide point-by-point responses to each concern. The modified content is marked in red.

****Journal Requirements:****

**Comment:** We noticed that you used the phrase 'unpublished data' in the manuscript. We do not allow these references, as the *PLOS* data access policy requires that all data be either published with the manuscript or made available in a publicly accessible database. Please amend the supplementary material to include the referenced data or remove the references.

**Response:** We sincerely appreciate the editorial team’s vigilance in ensuring compliance with *PLOS*’s data policies. In response to the concerns regarding references to "unpublished data," we have made the following amendments:

1. The sentence "We previously discovered that IL-9 production was increased in the sera of sporotrichosis patients caused by *S. globosa* (unpublished data)" (original lines 448-449) has been removed from the Discussion section. We cannot provide the relevant data because it will be used in another independent article. We have removed all related content to comply with journal policies on dual publication and ensure the independence of both studies.

2. The reference to murine model findings—"we observed Th17 dominant responses in the murine *S. globosa* footpad infection model (unpublished data)" (original lines 495–498)—has been replaced with newly generated experimental data, which are now included as supplementary material designated as S5_Fig (already uploaded with the revised submission).

**Reviewer 1**

**Comment 1:** Is public health relevance addressed? This study highlights the importance of IL-18 with a dual mechanism of action related to the regulation of Th1/Th2/Th17 immunity with a predominance of Th2 and which, through complex immunological mechanisms, allows local control of the disease. Although the study allows hypotheses to be established that may be relevant in future clinical and therapeutic studies, it is not yet possible to determine the precise relevance of the finding in public health.

**Response:** We appreciate the reviewer’s critical perspective on the public health implications of our work. Our study primarily investigates the immune response within *S. globosa*-infected skin lesions, with a focused analysis on the dual regulatory role of IL-18 in shaping local T helper cell responses (Th1/Th2/Th17) and its impact on disease chronicity, we agree that direct translation to population-level interventions requires further validation. While our current findings are mechanistic, they provide a roadmap for reducing sporotrichosis-related morbidity in endemic areas through precision immunomodulation.

**Comment 2:** The authors clearly outline the local and systemic immune mechanisms that influence the chronicity of sporotrichosis. I am satisfied with the experiments conducted, which have clarified that many of these mechanisms are similar (if not identical) to those reported in other research involving other species of *Sporothrix*.

**Response:** We are deeply grateful for the thorough and insightful evaluation of our work. Your insights have significantly strengthened the contextualization of our findings within *Sporothrix* immunopathology.

**Reviewer 2**

**Comment 1:** Is the sample size sufficient to ensure adequate power to address the hypothesis being tested? Uncertainty.

**Response:** We sincerely appreciate the reviewer’s critical inquiry regarding statistical power. Our study builds upon our prior work (Zu et al., Frontiers in Immunology. 2020, 11:570888), which revealed Th2 polarization in circulation of 68 sporotrichosis patients. While the current cohort for lesional skin analysis comprises 44 patients, the observed immune trends align robustly with our previous findings in peripheral blood. This consistency across sample types and cohorts strengthens confidence in the reproducibility of our conclusions. Although larger samples may further refine effect sizes, the statistical significance and mechanistic validation (e.g., antibody neutralization assays) collectively support the biological validity of our findings.

**Comment 2:** The length of "Introduction and Methods parts" is a bit too long, which should be polished.

**Response:** We thank the reviewer for the constructive suggestion. We have carefully revised both the Introduction and Methods sections to balance conciseness with scientific rigor. In the Introduction, we streamlined sentences, removed redundant content, and prioritized pivotal literature to sharpen the study’s rationale. In the Methods section, Table 1 (originally in the main text, containing demographic data of study participants) has been relocated to the Supplementary Materials and renumbered as S1 Table. Non-essential technical descriptions were removed to streamline the content, while critical parameters were retained. Additionally, methodological details were relocated to the Supporting Information to enhance the main text's conciseness and focus. We believe the updated text now provides a clearer and more focused presentation of the study’s context and experimental design, aligning with the journal’s emphasis on concise and accessible writing.

**Comment 3**: Tables 2 and 3: move these to supplemental material as they don't need to be in main text.

**Response**: Thank you for highlighting this issue. We have relocated Tables 2 and 3 to the Supporting Information (now labeled S2 and S3 Tables), as recommended. All relevant references to these data in the main text have been updated accordingly. This adjustment enhances the clarity and focuses of the core results presented in the main manuscript.

We have made all the suggested revisions in the manuscript, which is now uploaded as a marked-up file titled "Revised Manuscript with Track Changes.docx" and a clean version titled "Manuscript.docx." Additionally, all figure files have been processed through PACE and are compliant with *PLOS* requirements.

We believe the revised manuscript has been significantly improved thanks to the reviewers’ valuable suggestions. Should you require further clarification or additional changes, please do not hesitate to contact us.

Thank you again for your time and expertise. We look forward to hearing from you soon.

Sincerely,

Yu Zhen
